# Valproic Acid Downregulates RBP4 and Elicits Hypervitaminosis A-Teratogenesis—A Kinetic Analysis on Retinol/Retinoic Acid Homeostatic System

**DOI:** 10.1371/journal.pone.0043692

**Published:** 2012-09-19

**Authors:** Chao-Ming Chuang, Chi-Huang Chang, Hui-Er Wang, Kuan-Chou Chen, Chiung-Chi Peng, Chiu-Lan Hsieh, Robert Y. Peng

**Affiliations:** 1 Department of Nursing, Hungkuang University, Shalu County, Taichung City, Taiwan; 2 Research Institute of Biotechnology, Hungkuang University, Shalu County, Taichung City, Taiwan; 3 Department of Food and Applied Technology, Hungkuang University, Shalu County, Taichung City, Taiwan; 4 Department of Urology, School of Medicine, College of Medicine, Taipei Medical University, Taipei, Taiwan; 5 Department of Urology, Shuang Ho Hospital, Taipei Medical University, Zhonghe, Taipei, Taiwan; 6 Graduate Institute of Clinical Medicine, College of Medicine, Taipei Medical University, Xin-Yi District, Taipei, Taiwan; 7 Graduate Institute of Biotechnology, Changhua University of Education, Jin-De Campus, Changhua, Taiwan; University of South Florida College of Medicine, United States of America

## Abstract

**Background:**

Valproic acid (VPA) is an antiepileptic and anti-migraine prophylactic drug. VPA exhibits two severe side effects, namely acute liver toxicity and teratogenicity. These side effects are usually seen at the genetic and somatic levels. The cited action mechanisms involve inhibition of histone deacetylase, hypofolatenemia, hyperhomocysteinemia, and reactive oxidative stress. The proteomic information associated with VPA teratogenicity is still unavailable. We hypothesized that proteomic analysis might help us identify functional proteins that could be relevantly affected by VPA, and this phenomenon could be very sensitive in early embryonic stage, resulting in VPA teratogenicity.

**Methodology/Principal Findings:**

Proteomic analysis on the chicken embryos at Hamburger and Hamilton (HH) stage 28 showed that there were significant downregulations of ovotransferrins, carbonic anhydrase-2, retinol binding protein-4 (RBP4), NADH cytochrome b5 reductase 2 (CYB5R2), apolipoprotein A1, and protein SET, together with upregulation of 60S ribosomal protein L22. Among these, RBP4 was the most significantly downregulated (−32%). Kinetic analysis suggested that this situation could trigger hypervitaminosis A (+39.3%), a condition that has been well known to induce teratogenesis..

**Conclusions/Significance:**

This is the first report showing that VPA dowregulates RBP4. Our finding not only has led to a possible mechanism of VPA teratogenesis, but also has initiated new preventive strategies for avoiding VPA teratogeneis.

## Introduction

**Figure 1:**
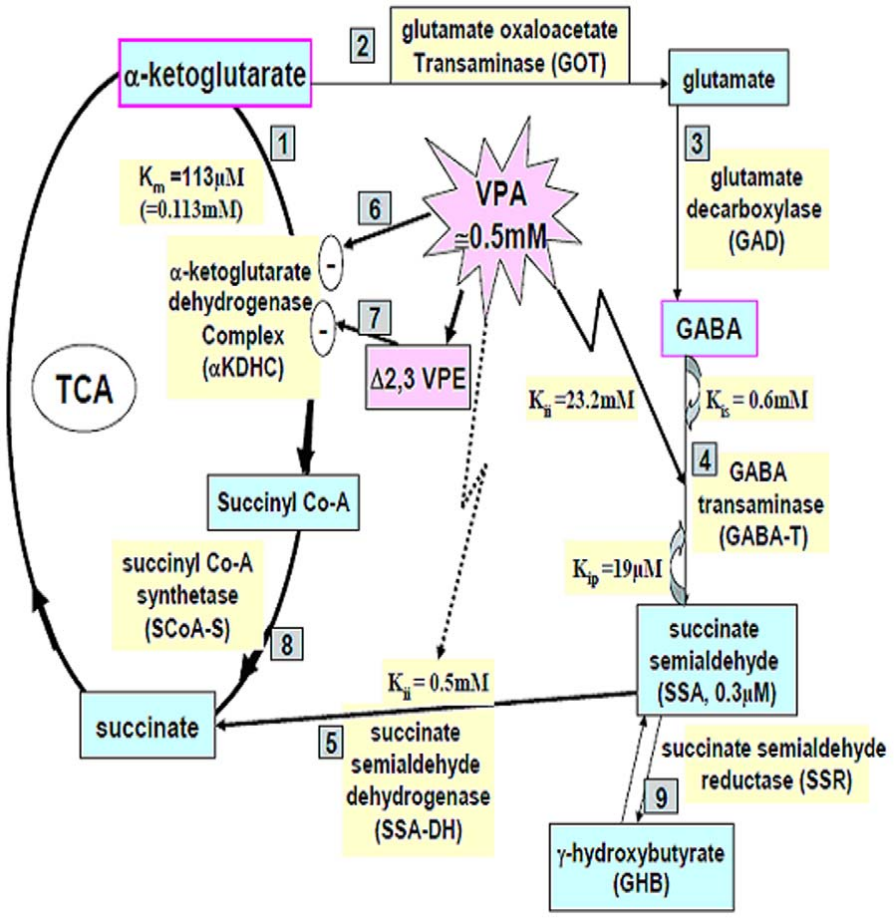
Summary of the pharmacological mechanism of action of valproic acid.

Valproic acid (VPA, 2-propylpentanoic acid) is an antiepileptic and anti-migraine prophylactic drug. VPA has anticonvulsive activity *via* inhibition of the citric acid cycle and elevation of γ-aminobutyric acid (GABA) levels in central nervous system (CNS) [1], [2]. Specifically, α-ketoglutrate (α-KGA) is generated from the TCA cycle and may be further transformed into other metabolites through two possible ways: one passes through succinyl CoA to produce succinic acid by action of α-ketoglutrate dehydrogenase complex (αKDHC) (reaction 1) and succinyl CoA synthetase (reaction 6) [1], [2], while the other takes the way from α-KGA to glutamate (reaction 2) then to GABA (reaction 3) and succinate semialdehyde (SSA) (reaction 4), and finally to succinate (reaction 5) (Fig. 1) [2].

Most clinical physicians start treating patients with 250–750 mg of VPA per day. The dosage is then steadily increased by 5 to 10 mg/kg per week until seizures stop or the patient experiences too many side effects. Most patients take between 750 to 3,000 mg (1.13–4.51 mM) each day, but divided into two or three doses. Children may require higher doses and the elderly usually need lower doses. The therapeutic blood level of sodium valproate (MW: 166.19) is generally considered to be 50–100 µg/mL (0.3–0.6 mM; lower for the elderly), but adjustments depend on the clinical response (Epilepsy.com/Professionals). Administration of VPA 400 mg/kg in mice gives a concentration of VPA approximately 0.5 mM in brain (Fig. 1) [3], indicating that VPA has relatively high transportability across the brain blood barrier (BBB). Chronic administration of VPA in therapeutic doses to dogs produces intracerebrally approximately 0.5–2.0 µM of 2-*n*-propylpent-2-enoic acid (Δ2,3 VPE) (a metabolite of VPA) (Fig. 1) [4]. Pharmacologically, VPA tends to interfere with the tricarboxylic acid cycle (TCA cycle) and increase gamma-aminobutyric acid (GABA) production [1]. Nonetheless, at 20 mM or higher doses, VPA does not show any direct effect on αKDHC [1] (Fig. 1). Rather, CoA derivatives of VPA and Δ2,3 VPE (substrate inhibition constant K_is_ = 41 µM) exert competitive inhibition on the αKDHC (reaction 1) (Fig. 1) with Δ2,3 VPE CoA causing mixed type inhibition [1]. Citric acid cycle flux is thus reduced, alternatively the flux into GABA synthesis is increased, which pertinently explains the anticonvulsant activity of VPA as well as part of its toxicity.

However, it should be noted that the inhibitor inhibition constants (K_ii_) of VPA for GABA transaminase (GABA-T) and SSA dehydrogenase (SSA-DH) are 23.2 and 0.5 mM, respectively (reactions 4 and 5) (Fig. 1) [3]. The relationship of these two values to the cerebral VPA level (≅0.5 mM) and the K_ii_ of SSA-DH (0.5 mM) implies that SSA-DH is more likely to be the therapeutic target of VPA (Fig. 1). In addition, because the substrate inhibition constant (K_is_) of GABA for GABA-T is 0.6 mM [3], suggesting that when VPA is to be used as an anticonvulsant, the minimum effective serum concentration has to be raised to ≥0.6 mM (reaction 4). Normally the intracerebral SSA reaches 0.3 µM and the feedback inhibition of SSA on GABA-T could occur (reaction 4, Fig. 1) provided that VPA has effectively inhibited SSA-DH (K_ii_ = 0.5 mM) and simultaneously the SSA level has been raised to the product inhibition constant (K_ip_), i.e. ≥19 µM [3].

Clinically, VPA exhibits two severe side effects, namely acute liver toxicity and teratogenicity [5], which are mostly seen at the genetic and somatic levels [6]. The latter involves a diversity of physiopathological changes including the neurotubular deficits (NTD) [7], [8]. These deficits have been ascribed to i) disruption of folate or homocysteine metabolism [2], [9], [10]; ii) the induction of oxidative stress [11]; iii) the presence of oxidative stress leading to ω- and β-oxidation [12], [13]; and iv) an inhibitory effect on histone deacetylase (HDAC) [14], [15], [16].

We hypothesize the teratogenic changes induced by VPA could occur in early stage of embryo, in parallel some functional proteins can be altered. Such changes might provide useful clues to help us explaining VPA teratogenicity and exploiting the preventive strategies. In this present study, we performed a proteomic analysis on 5.5-day HH stage 28 chicken embryos. The discriminating level of significance was set at ±30% (*p*<0.01). By referring to some cited biochemical parameters, we mathematically deduce the outcome that might be elicited by such a big change of the specifically relevant protein.

## Materials and Methods

### Chemicals

Bovine serum albumin (BSA), sodium dodecyl sulfate (SDS), glycerol, bromophenol blue, Coomassie Brilliant Blue R (CBR), iodoacetamide (IAA), silver nitrate, N,N,N′,N′-tetramethyl-ethylenediamine (TEMED), Tween-20, ammonium bicarbonate (NH_4_HCO_3_), and potassium ferricyanide were purchased from Sigma Co. (St. Louis, MO, USA). Methanol, ethanol, and n-butanol were provided by E. Merck Co. (Damstadt, Germany). Bio-Rad protein assay kits, Bio-Rad protein standards solutions, amphoteric solutions (Bio-Lyte 3–10 Ampholyte), 30% bisacrylamide, 19∶1, and mineral oil were products of Bio-Rad (Hercules, California, USA). The 2-D Clean-up Kit was a product of GE Healthcare Bio-Science Corp. (Piscataway, USA). Hydrochloric acid, sodium chloride, potassium chloride, monopotassium bihydrogen phosphate, and disodium hydrogen phosphate were manufactured by Wako Pure Chemicals (Tokyo, Japan). Glycerol, urea, CHAPS, and DL-dithiothreital were purchased from Bio-Basic Inc. (NY, USA). The anti-rabbit IgG HRP-linked antibodies, Tris buffered saline with Tween-20 (TBST-10X), and cell lysis buffer were provided by Cell Signaling Technology Co. (Massachusetts, USA). ProteoJET™ Mammalian Cell Lysis Reagent, ProteoBlock™ and Protease Inhibitor Cocktail were products of Fermentas Co. (USA). Trypsin was a product of Promega (Madison, Wisconsin, USA). Sodium thiosulfate pentahydrate, sodium carbonate and acetonitrile (ACN) were supplied by JT-Baker Chemical Products Trading (Shanghai) Co., Ltd.

### Preparation of reagents

Phosphate buffered saline with Tween-20 (PBST) was prepared by dissolving 8 g NaCl, 0.863 g Na_2_HPO_4_, 0.2 g KCl, and 0.2% Tween-20 in 800 mL deionized water and the pH was adjusted to 7.4, finally DW was added to make up 1 L. Tris buffer saline with Tween-20 (TBST) was prepared by dissolving 8 g NaCl, 2.42 g Tris base, and 0.1% Tween-20 in 800 mL DW, pH adjusted to 7.6, then made up to 1 L with deionized water. SDS-PAGE electrophoresis buffer (Running buffer) was prepared by dissolving 3.03 g Tris-base, 14.41 g glycine, 1 g SDS in 800 mL deionized water, the pH was adjusted to 8.3 and deionized water was added to make up 1 L. Transfer buffer was prepared by dissolving 6 g Tri-base, 28.8 g glycine, 2 g SDS, and 500 mL methanol. The final volume was made up to 2 L by deionized water. Destaining buffer was prepared by mixing 10% acetic acid, 30% methanol and 60% double distilled water. CBR staining buffer was prepared by dissolving 1.5 g Coomassie Blue-R, 250 mL methanol, and 50 mL acetic acid in deionized water to make a final volume 250 mL. Solution A was prepared by dissolving 0.8 g bis-(N, N-methylene)-acrylamide, and 30 g acrylamide with deionized water to make up a final volume 100 mL. Solution B was prepared by dissolving 18.4 g Tris-base in deionized water 50 mL. 0.23 mL TEMED was added and the pH was adjusted to 9.0, deionized water was added to make up the volume to 100 mL. Solution C was prepared by dissolving 5.98 g Tris-base, deionized water 50 mL. TEMED 0.46 mL was added, pH was adjusted to 6.7, and deionized water was added to make up 100 mL.

### Source of fertilized eggs and embryo treatment

Thirty two day-1-fertilized Leghorn eggs were supplied by Qing-Dang Chicken Farm (Taichung, Taiwan). The fertilized eggs were placed in an incubator (Haw-Yang Agricultural Farm, Taichung, Taiwan) and incubated at 37°C, relative humidity 50–60%, for 1.5 days. Previously, we have showed the optimum teratogenic dosage of valproic acid to be 0.6 mM [17] and the same dosage was applied in this experiment. Briefly, the fertilized eggs were moved to a laminar flow chamber. A hole with the size 2 mm×2 mm was aseptically drilled through the egg shell using a hole-driller. The embryos were gently moved as close as possible to the hole opening by carefully turning the egg between the observer's eyes and a direct strong light source; once proximity of the embryo was achieved, 100 µL of VPA (30 mM) in PBS or PBS per egg was injected using a tip injector. After injection, the openings were aseptically sealed with 3 M tape and the incubation was continued. The sampling point was set to be day 5.5 HH stage 28. Two embryos were frozen immediately for Hematoxylin-Eosin staining. Ten embryos were pooled by breaking their shells, rinsed twice with PBS (4°C), and homogenized to produce the embryonic homogenate samples (EHSs) for assaying HDAC, SOD, and hydrogen peroxide. The protein content of the EHSs was analyzed according to the manufacturer's instruction (Bio-Rad, Hercules, California, USA). The remaining twenty embryos were pooled and used for protein extraction as described in section 2.7.

### Assay for histone deacetylase (HDAC)

Histone deacetylase activity was determined according to Kwon et al., (2002) [18]. In brief, 0.5 mL of cold lysis buffer (pH 7.5, containing 10 mM Tris-HCl, 10 mM NaCl, 15 mM MgCl_2_, 250 mM sucrose, 0.5% Triton-X100, and 0.1 mM EGTA) was added to homogenized EHS (100 mg) and the mixture homogenized. After agitating for 10 s, the mixture was left to stand at 4°C for 15 min. A total of 1.6 mL of the mixture was mixed well with a cold sucrose cushion (containing 30% sucrose, 10 mM of pH 7.5 Tris-HCl, 10 mM NaCl, and 3 mM MgCl_2_), and centrifuged at 1300×g at 4°C for 10 min. The supernatant was discarded and 400 µL of 10 mM Tris-HCl buffer (pH 7.5, containing 10 mM NaCl) was added to the residue,. The pellets were dispersed and the mixture was centrifuged at 1300×g at 4°C for 10 min. The supernatant was then discarded and 150 µL of Extraction Buffer (pH 7.5, containing 50 mM HEPES, 420 mM NaCl, 0.5 mM EDTA, 0.1 mM EGTA, and 10% glycerol) was added to the residue. The mixture was agitated to resuspend the pellets. After ultrasonication for 1 min, the mixture was centrifuged at 10000×g at 4°C for 10 min. The supernatant was removed and stored at −80°C until use. To determine histone deacetylase activity, four supernatant samples of 10 µL were transferred into a 96-well plate. Next 160 µL of assay buffer was added to two of the four samples to serve as sample group A. In parallel, 150 µL of assay buffer and 10 µL Trichostatin A solution were added to the other two samples to serve as sample group B. Next, 10 µL HDAC substrate solution was added to each of the four samples. After agitated at 37°C for 30 min, 40 µL of developer agent was added and the mixture left to react at ambient temperature for 15 min. The fluorescence intensity of the final solution was read with an ELISA Fluorescence Reader at emission wave length 440–465 nm after excitation at wavelength 340–360 nm. To establish the calibration curve, a deacetylated histone standard (2.1 mM) was diluted 10-fold initially and then diluted to 10, 21, 42, 84, and 168 µM, respectively. To 10 ul of each standard solution, 160 µL assay buffer and 10 µL HDAC substrate solution were added and the assay was conducted as described above. The amount of HDAC was calculated from the calibration curve according to the following equation:
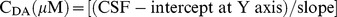
where CSF = F_B_−F_A_.

C_DA_ (µM) = the concentration of deacetylated compound (µM).

F_B_: the fluorescence intensity obtained from sample group B.

F_A_: the fluorescence intensity obtained from sample group A.

The original HDAC activity was calculated from
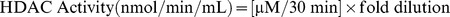



### Assay for superoxide dismutase (SOD)

Mattiazzi et al. (2002) [19] was followed to determine the superoxide dismutase activity. HEPES buffer (20 mM) (containing 1 mM EGTA, 210 mM mannitol, and 70 mM sucrose) was added to 100 mg of EHS, the mixture was then homogenized, and centrifuged at 14000×g at 4°C for 5 min. Radical detector (200 µL) was added to the supernatant and mixed well by gentle twisting of the tube. Next, 20 µL of diluted xanthine oxidase solution was added to the mixture and after gentle shaking the mixture was left to stand for 20 min. The optical density was read at 450 nm by ELISA reader.

### Assay for hydrogen peroxide (HPO) in embryos

The hydrogen peroxide level in embryo was assayed using the protocol of Nourooz-Zadeh et al. (1994) [20]. Briefly, 100 µL of PBS were added to 100 mg of EHS and the mixture homogenized and centrifuged at 12000×g. Samples (25 µL) of supernatant were loaded onto a 96 well plate, 250 µL of color reagent was added to each well and left to react for 30 min at ambient temperature. The optical density was read at 620 nm with an ELISA reader. A calibration curve was created using hydrogen peroxide solution (Merck, Germany) at concentrations of 5, 10, 20 30, 40, 50 and 60 µM using the same procedure.

### Protein extraction

This was done by following the manufacturer's instructions. Briefly, sample embryos (EMB) were diluted two-fold using cell lysis buffer and the mixture homogenized and stored on ice. After centrifugation at 14000×g, the supernatant was transferred to microcentrifuge tubes and stored at −80°C until use.

### 2D-electrophoresis

#### 2D-clean up

The supernatant after protein extraction (3 mg) was transferred to a 1.5 mL tube and 300 µL of precipitant was added with agitation for 4–5 s. After standing at 4°C for 15 min, co-precipitant (300 µL) was added. The mixture was agitated and then centrifuged at 8000×g at 4°C for 10 min. The supernatant was carefully removed and the residual pellet was re-centrifuged at 8000×g at 4°C for 5 min to remove remaining supernatant. Next 40 µL of double distilled water was added to the pellet and the proteins dispersing using a thin spatula followed by ultrasonicated for 1 min. Wash buffer (1 mL), previously cooled to −20°C, and 5 µL of additive solution, were then introduced into the mixture. Next, the mixture was agitated at −20°C at 10 min intervals for 30 min followed by keeping the solution at −20°C for 30 min. The solution was then centrifuged at 8000×g at 4°C for 10 min and the pellet blown to dryness for 5 min. The residue was redissolved in 420 µL rehydration buffer containing DDT at ambient temperature for 30 min and centrifuged at 8000×g for 10 min to remove the any insoluable material. An aliquot of the supernatant (350 µL) was used to carry out the IEF electrophoresis.

#### IEF electrophoretic analysis

The strip holder was rinsed with neutral detergent to remove the protein residues left behind from previous work, then washed twice with double distilled water and left to dry at ambient temperature. The protein sample prepared as described above (3 mg) was spread evenly onto the strip holder (Hoefer TM TE22). Two filter pads after wetted with double distilled water were placed onto the two electric terminals on the focusing tray. The protective covering membrane on the IPG strips (immobilized pH gradient strip), which had previously refrigerated at −20°C, was removed. The IPG strips were fixed face down on to the focusing strip. Then 2.5 mL of mineral oil (IPG Cover Fluid) was placed onto the IPG strip to avoid the evaporation of the rehydration buffer and the focusing tray was covered. The whole focusing tray was placed onto a Protean IEF cell (Bio-Rad Labratories Inc.) (California, USA). The IEF electrophoresis was conducted at 20°C. The subsequent protocols involved rehydration at 50 V for 12 h and conditioning at 250 V for 15 min, followed by raising the electric potential up to the focusing voltage. During IEF, IPG strips that were 7 cm long were focused at a voltage of 4000 V for a V*h of 20000 V*h. For IPG 11 cm and 17 cm, the corresponding values were 8000 V and 40000 V*h, and 10000 V and 6000–8000 V*h, respectively. After focusing, the voltage was set at 500 V to avoid the further diffusion of proteins already focused.

#### SDS-PAGE

After IEF electrophoresis, the IPG strips were rinsed with double distilled water to remove the residual mineral oil. Fresh balancing fluid (containing DDT) was added for 20 min to allow equilibration, then the fluid was removed and more balancing fluid (containing IAA) was added and the strips left to equilibrate for 20 min. The equilibrated IPG strips were then placed onto a prepared 10% SDS-PAGE gel with tight contact. Then 0.5% agarose solution was used to cover and fix the strips. The gel was placed in either a MiniProtean II cell or a Protean II xi 2-D cell. After the protein marker (5 µL) had been added, the electrophoresis was begun using the conditions: 4°C, starting conditions of 16 mA/gel for 30 min and then 35 mA/gel for up to 5 h until the dye had reached the bottom of the gel. For each 2D analysis, at least six replicates were performed to assure the accuracy.

#### Silver staining

The SDS-PAGE was removed from the apparatus and silver stain carried out as follows: i) fixation for 30 min with a fixation agent (95% ethanol 210 mL, acetic acid 50 mL, and double deionized water to make up to 1000 mL); ii) sensitizing for 30 min with an sensitizing solution (95% ethanol 158 mL, sodium thiosulfate 2.44 g, sodium acetate 34 g, and double deionized water to make up to 500 mL; iii) washing with double deionized water six times, each time for 5 min; iv) silver staining for 20 min with staining solution (500 mL of 0.25% silver nitrate solution containing 200 (L of 37% formaldehyde); v) rinsing twice with double deionized water, each time for 1 min; vi) developing with developing solution (sodium carbonate 12.5 g, 100 (L of 37% formaldehyde, and double deionized water to make up to 1 L); and vi) halting the reaction with stopping fluid (acetic acid 25 mL and double deionized 475 mL). The developed SDS-PAGE was scanned using Melanie 7 software to pinpoint protein spots that were significantly different (p<0.05).

### Identification of proteins by LC-MS/MS

The protein spots pinpointed as different between the treated and untreated day-5.5 embryos were analyzed by LC-MS/MS (Mass Spectrometry: Bruker Daltonics, HCT Ultra PTM Discovery System; HPLC: Dionex, UltiMate 3000. Courtesy of The Proteomic Center of China Medical University). Briefly, the finished SDS-PAGE was rinsed twice with mine Q water wash, each time for 10 min. Using clean cutting blades the SDS-PAGE gels were cut into pieces to separate the protein spots of interest and the gel pieces were transferred into a microcentrifuge tube; then 200 (L of wash solution was added and the mixture agitated for 15 min, after which the supernatant fluid was removed. Destain solution (200 (L) was added and agitated for 5 min, and the supernatant was removed. ABC solution (10 mM; 200 (L) was added, the mixture was agitated for 5 min and the supernatant removed. The treatment with ABC solution was repeated for several times. For dehydration, acetonitrile anhydrous (ACN) (200 (L) was added and agitated for 5 min. The dehydration process was repeated until the color of gel turned to whitish and became curled flakes, at which point the supernatant was removed. Then a SpeedVac was used for 10–20 min to completely remove all ACN. A total of 100 (L of reduction solution was then added to the residue and the reaction allowed to proceed at 56°C for 15 min. After cooling, the supernatant was removed and alkylation solution (100 (L) was added; the mixture was allowed to react for 20 min avoiding the direct sun light after which the supernatant was removed. The residue was washed with wash solution (200 (L) for 15 min and the supernatant removed. ACN dehydration agent (200 (L) was then added to the residue again and dehydration continued until the residue turned into whitish and committed curing flakes. At this point the supernatant was removed using Speed Vac for 10–20 min to completely remove the ACN. Enzyme solution (trypsin 1 ng/(L, 2 (L) was then added to the residue and the digestion was carried out at 4°C for 1 h. After digestion, ABC (10 mM, 10 (L) was added, and the mixture was left to further reaction for 24 h at 40°C. The reaction mixture was then subjected to gradient extraction using 30 (L 40–70% ACN/0.1% formic acid with ultrasonication for 10 min. After filtered with a Z-tip, the extracts were dried under vacuum. Finally, the dried residue was analyzed using LC-MS/MS. The LC-MS/MS was performed with a nanoflow LC system (Ultimate 3000 NanoLC system, Dionex) coupled to an ion trap mass spectrometer (HCTultra PTM Discovery system, Bruker, Germany). The sample was injected into a trap column (Acclaim PepMap C18, 5 µm, 1×5 mm, Dionex, The Netherlands) and separated online using a reverse phase column (Atlantis DC18, 3 µm, 75 µm×150 mm, Waters, Milford, MA) at the flow rate of 0.25 µL/min using a 58 min 5–50% acetonitrile/water gradient and 15 min 70–90% acetonitrile/water gradient. Acquisition were performed in THE data-dependent Auto-MS/MS mode with a scan range of 100–2400 m/z, and four precursor ions selected from the MS scan range of 421–1500 m/z. The charge state was set to 2+ and 3+. Before each sample analysis, 50 fmol of tryptic BSA standard was used to confirm the column efficiency and LC-MS sensitivity. For the MASCOT search, the data was deisotoped and converted by the Data Analysis Version 4.0 (Build 275). The search parameters of MASCOT for peptide and MS/MS mass tolerance were ±0.5 Da and ±0.5 Da, respectively, with allowance for one missed cleavage in the trypsin digest. Search parameters were selected as Taxonomy–*Metazoa (Animals)*; enzyme–trypsin; fixed modifications–carbamidomethyl (C); and variable modifications–oxidation (M). Peptides were considered as identified if their MASCOT individual ion score was higher than the MASCOT score 30 (*p*<0.001).

### Statistical analysis

The dataset obtained was analyzed using Statistical Analysis System 9.0 (SAS 9.0) and expressed as mean ±SD from triplicate experiments. The variance between groups was analyzed using Duncan's Multiple Range Test. A level of *p*<0.05 was set as the confidence level.

## Results

### Malformed individual and its damaged cervical muscle

**Figure 2:**
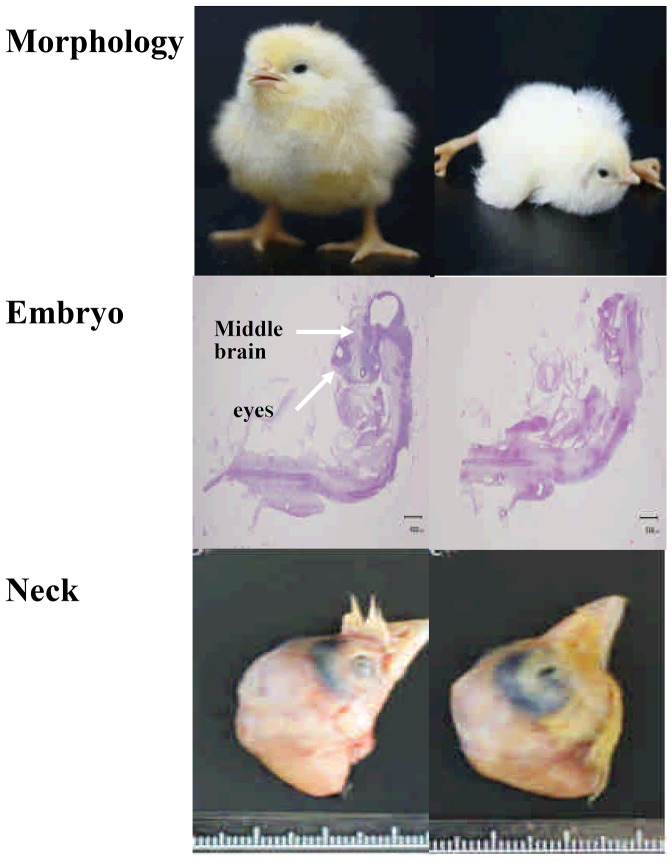
Comparison of the normal control chicks and malformed chicks after VPA treatment. The morphology of the normal control chicks (upper left panel) and the malformed VPA treated chicks (upper right panel). A light micrograph of a normal day 5.5-day embryo 20× (middle left panel), and of a malformed day 5.5 day-embryo 20× (middle right panel) after hematoxylin-eosin (H&E) staining. Neck muscle: neck with a normal cervical muscle (lower left panel) and neck with a malformed cervical muscle using edema and inflammatory fibrotic tissue (lower right panel). Normal chick: PBS treated. Malformed chick: treated with VPA (30 mM) 100 µL/fertilized egg.

Some of the major morphologically malformed changes induced by VPA are shown in Fig. 2. The right column shows the characteristics of malformed chicks including the loss of the ability to stand upright (upper right panel) and incomplete development of the midbrain in the 5.5-day embryo (20× middle right panel) using hematoxylin-eosin (H&E) staining (middle right panel). In addition the neck muscles were severely affected by VPA with edema and inflammatory fibrosis of the cervical muscle (lower right panel).

### HDAC and superoxide dismutase were significantly inhibited in parallel with the overproduction of hydrogen peroxide

HDAC activity was downregulated by 54.5%, while SOD activity was downregulated by 21.1%. In parallel to this, the production of hydrogen peroxide was significantly increased by 28.3% (Table 1).

**Table 1:** Changes in the level of HDAC, SOD, and hydrogen peroxide after treatment with valproic acid.

Item	Level		
	control	VPA-treated	% change
HDAC, nmol/mg protein	2.2±0.4	1.0±0.3	−54.5±2.6
SOD,U/mg protein	0.57±0.06	0.45±0.02	−21.1±1.4
Hydrogen peroxide, nmol/mg protein	6.0±0.8	7.7±1.5	+28.3±2.3

100 µL of valproic acid (30 mM) per egg (approximately 60 mg/kg) was applied.

### The proteomic analysis revealed changes in ten functional proteins

**Figure 3:**
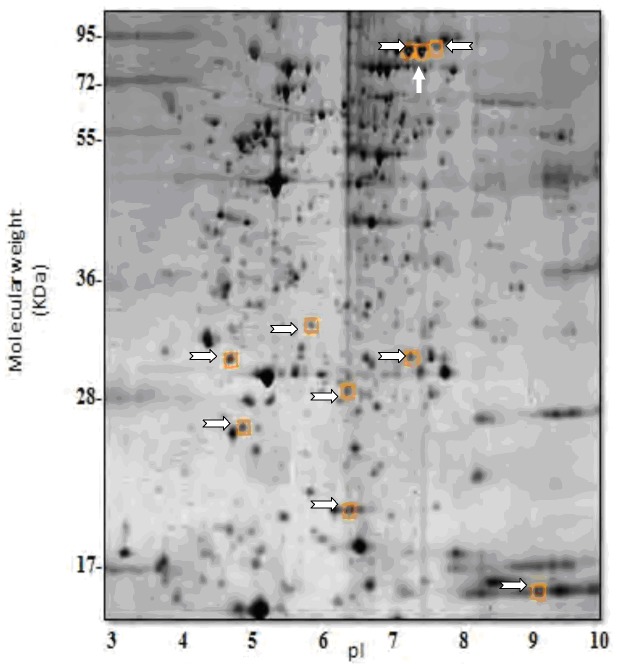
Silver staining of proteins obtained from day 5.5 chick embryo and analyzed by 2-D electrophoresis. The electrophoretic pattern of the isolated proteins was analyzed with Melanie 7. Comparing with the PBS control proteins, the built-in statistical software identified ten protein spots as being significantly different (*p*<0.05). The spots were numbered 24, 33, 48, 51, 58, 141, 146, 233, 258 and 275. The fold difference and *p*-value for each protein were determined and are listed in Table 5. Each spot was identified by LC-MS/MS and the results were compared with the MASCOT database.

The proteomic analysis revealed that the protein levels of ten functional proteins were significantly altered in the day-5.5 HH stage 28 embryos after VPA treatment (Fig. 3). The Mr[Da]/PI (SSP; MASCOT score) values for these proteins were: 22843/5.93 for retinol-binding protein-4 (RBP4) (24; 158); 32132/4.13 for Protein SET (SET) (33; 105); 30661/5.58 for Apolipoprotein A-1 (APOA1) (48; 134); 29388/6.56 for carbonic anhydrase II (CA-II) (51; 569); 33607/9.41 for NADH-cytochrome b5 reductase 2 (CYB5R2) (58; 30); 79551/6.85 for ovotransferrin (LTF) (141; 897); and 14777/9.21 for 60S ribosomal; protein L22 (RPL22) (275; 89) (Table 2). The percentage coverage for RBP4, SET, APOA1, and CA-2 was 16%, 3%, 18%, 29%, 10% respectively (Table 2). The ovotransferrins had the largest percentage coverage and were the most abundant of the peptides at 32%, 37%, and 24% coverage with MASCOT scores of 897, 1171, and 643, respectively. RPL22 had a modest coverage of 21% (Table 2).

**Table 2:** Changes in the functional proteins in embryos after treatment with valproic acid.

SSP	Protein name	Gene	Theoretical Mr [Da]/pI	MASCOT score	SwissPort accession number	Coverage [%]	No. of peptides
24	Retinol-binding protein 4	RBP4	22843/5.93	158	P41623	16%	7
33	Protein SET	SET	32132/4.13	105	Q5F3R9	3%	1
48	Apolipoprotein A-1	APOA1	30661/5.58	134	P08250	18%	4
51	Carbonic anhydrase 2	CA2	29388/6.56	569	P07630	29%	7
58	NADH-cytochrome b5 reductase	CYB5R2	33607/9.41	30	Q5ZHX7	10%	2
141	Ovotransferrin	LTF	79551/6.85	897	P02789	32%	19
233	Ovotransferrin	LTF	79551/6.85	1171	P02789	37%	28
258	Ovotransferrin	LTF	79551/6.85	643	P02789	24%	15
275	60S ribosomal protein L22	RPL22	14777/9.21	89	Q98TF8	21%	4

Matching peptides vs. total number of peptides submitted to database search, sequence coverage and scores obtained by peptide fingerprinting matching are listed. Protein accession number and theoretical pI and Mr and protein functions were obtained by SwissPort and NCBI databases.

Image analysis using SSP software was used to assign spot numbers 24, 33, 48, 51, 58, 141, 146, 233, 258, and 275 and to pinpoint these proteins as having a significant change in protein level, namely *p* = 0.0090, 0.0261, 0.0085, 0.0466, 0.01976, 0.0391, 0.0111, 0.0184, 0.0243, and 0.034, respectively; this was followed by LC-MS/MS. All these proteins, with the exception of 60S ribosomal protein L22 (RPL22) were downregulated. The changes in expression (fold change/assigned number) were: (−1.32/24), (−1.16/33), (−1.18/48), (−1.14/51), (−1.17/58), (−1.10/141), (−1.27/146), (−1.12/233), (−1.27/258), and (+1.11/275), respectively (Table 3), which means the downregulation of protein 24 (RBP4) had easily attained the initially set level of discrimination (≥30%, *p*<0.0089) (Table 3).

**Table 3:** LC/MS/MS analysis and MASCOT score assignment of the functional proteins identified as being significantly affected by valproic acid treatment.

SSP	Fold change (VPA/PBS)	*p*-value	Identified proteins
24	−1.32	0.0089	Retinol-binding protein 4
33	−1.16	0.0261	Protein SET
48	−1.18	0.0085	Apolipoprotein A-1
51	−1.14	0.0465	Carbonic anhydrase 2
58	−1.17	0.0197	NADH-cytochrome b5 reductase
141	−1.10	0.0390	Ovotransferrin
146	−1.27	0.0111	unidentifiable protein
233	−1.12	0.0184	Ovotransferrin
258	−1.27	0.0243	Ovotransferrin
275	1.11	0.0340	60S ribosomal protein L22

Protein identification numbers were assigned by the image analysis software.

SSP are listed in the first column. Nine proteins were downregulated and one upregulated.

The *p*-values were tested by Student's *t* test from six independent experimental data (n = 6).

### Retinol binding protein 4 was the most significantly affected protein

The change in expression of retinol binding protein 4 (RBP4) or spot 24 was −1.32 fold, that is −32% (*p*<0.0089, Table 3). Based on the above finding, we have discussed RBP4 (protein number 24) below with respect to its probable relationship to VPA teratogenesis.

## Discussion

### Malformations induced by valproic acid

Clinically, VPA has been shown to cause immature development of cardiovascular system, incomplete osteogenesis and a neurotubular deficit [6], resulting in encephaly and lumbosacral meningomyelocele [10]. Similar malformations were found in the day-5.5 HH stage 28 chicken embryos (Fig. 2).

### Significant inhibition of HDAC implies high teratogenicity

HDACs are direct targets of VPA [21]. Formerly HDACs have been described as the teratogenic receptors involved in VPA-induced neural tube defects (NTDs) [5], [22]. HDACs deacetylate lysine residues on histone tails and condensed chromatin, which limits the access of transcriptional activators to the DNA [23]. This study shows that VPA inhibits HDAC activity (−54.5%) (Table 1), which is consistent with previous studies [11], [14], [15]. However, in another recent study, an increase of 6-fold in histone H3 acetylation (+20%) was noted after treatment with VPA [24]. To date, all tested histone deacetylase inhibitors (HDACi) have been shown to be teratogenic [14].

### VPA inhibits expression of superoxide dismutase resulting in reduced antioxidative capacity

Typical VPA toxicity involves the significant suppression of SOD activity and an overproduction of hydrogen peroxide. In the present study, SOD activity was suppressed (−21.1%) (Table 1), indicating that VPA reduces antioxidative defensive capacity [25]. It is worth noting that a reduction in SOD levels in itself does not produce malformation, but rather hydrogen peroxide and/or hydroxyl radicals are known to be responsible for malformation via the effects of superoxide anions (•O2^−^) [26].

### Overproduction of hydrogen peroxide could result in the production of nitric oxide free radical (•NO) and peroxynitrite (OONO^−^)

Hydrogen peroxide was increased by 28.3% when VPA was administered (Table 1). The production of hydroxyl free radical can result either in superoxide anion radicals producing hydroxyl free radical via H_2_O_2_ and SOD or in superoxide anion radicals reacting with nitric oxide (•NO) to give peroxynitrite (OONO^−^), which in turn produces nitric acid (HOONO) and thence hydroxyl free radical [25]. In terms of quantum chemistry, the reaction of superoxide with non-radicals is spin forbidden. Hence in biological systems, its main reactions are with itself (dismutation) or with another biological radical such as nitric oxide (NO) or a metal. The superoxide anion radical (•O_2_^−^) spontaneously dismutates to O_2_ and H_2_O_2_ quite rapidly (∼10^5^ M^−1^ s^−1^ at pH 7). Physiologically, SOD is required because superoxide is able to react with a number of sensitive and critical cellular targets. For example, superoxide reacts with NO radicals (•NO) to produce toxic peroxynitrite (OONO^−^), which can be very detrimental to many biomolecules including DNA, RNA, enzymes, and a diversity of signaling peptides [27]; the result is an indirect oxidative attack by VPA on DNA, RNA and proteins. In addition, peroxynitrite spontaneously and rapidly is converted into nitric acid, which in turn decomposes to yield hydroxyl free radicals and nitrogen dioxide free radicals [25]. Obviously, in our experiment, the overproduction of hydroxyl free radicals (•OH) after treatment with VPA did not occur via the dismutation of superoxide anion radicals (•O_2_^−^) by SOD as demonstrated by Ďuračková (2010) [25]. Thus, restoring SOD activity via treatment with a nutraceutic might be an effective strategy, but the effectiveness would depend greatly on *in situ* biological circumstances.

Kinetically, the dismutation rate of superoxide anion radical (•O2^−^) is second order and the decomposition rate of superoxide by SOD is first order with respect to initial superoxide concentration [27]. At high concentrations, the half-life of superoxide is very short (e.g., 0.05 seconds), but the half-life is quite long at low concentrations, namely up to 14 hours. Moreover, SOD has the largest k_cat_/K_M_ (an approximation of catalytic efficiency) of any known enzyme (∼7×10^9^ M^−1^ s^−1^) [27], implying that the reaction rate is “diffusion limited” even at subnanomolar concentrations. Specifically, superoxide is still able to inactivate the citric acid cycle enzyme aconitase at such concentrations, poisoning energy metabolism and releasing potentially toxic iron. Aconitase is one of several iron-sulfur containing (de)hydratases that are part of various metabolic pathways and known to be inactivated by superoxide [28]. Since VPA decreases SOD activity (Table 1) and the downregulation of RBP4 is likely to decrease NO production [29], if this is true then an effect by VPA on aconitase and hence the citric acid cycle is unlikely.

### RBP4 is the most significantly altered enzyme in the presence of VPA and this results in a diversity of biological effects

Ten functional proteins were found significantly altered in day-5.5 HH stage 28 embryos by VPA treatment. Among these, RBP4 was found to be the most significantly downregulated (−32%, *p*<0.0089) (Table 3). RBPs play roles in the regulation of retinoid metabolism, and they are widely expressed in the developing embryo [30], [31], [32]. RBP4 affects cardiomyocyte differentiation [33]. They are released from the liver and act as cytoplasmic carriers of lipophilic retinoids [34]. Upregulation of RBP4 significantly increases p-Akt and p-eNOS production as well as inhibiting p-ERK1/2 production [29]. RBP4 has a robust and acute effect whereby it increases NO production, which results in vasodilatation [29]. It is worth noting that expression of N-cad, collagen IV (col-IV), c-jun, RBP, bcl-2, RAR alpha, TGF-β2, EMX-2, and PAX-3 during the embryonic development are timepoint-dependent events and all these factors have systemic sequential effects on neutral tube closure (NTC) [35]. However, whether the downregulation of RBP4 is a direct or indirect effect of VPA remains unclear.

### RBP4 is involved in retinoid metabolism

**Figure 4:**
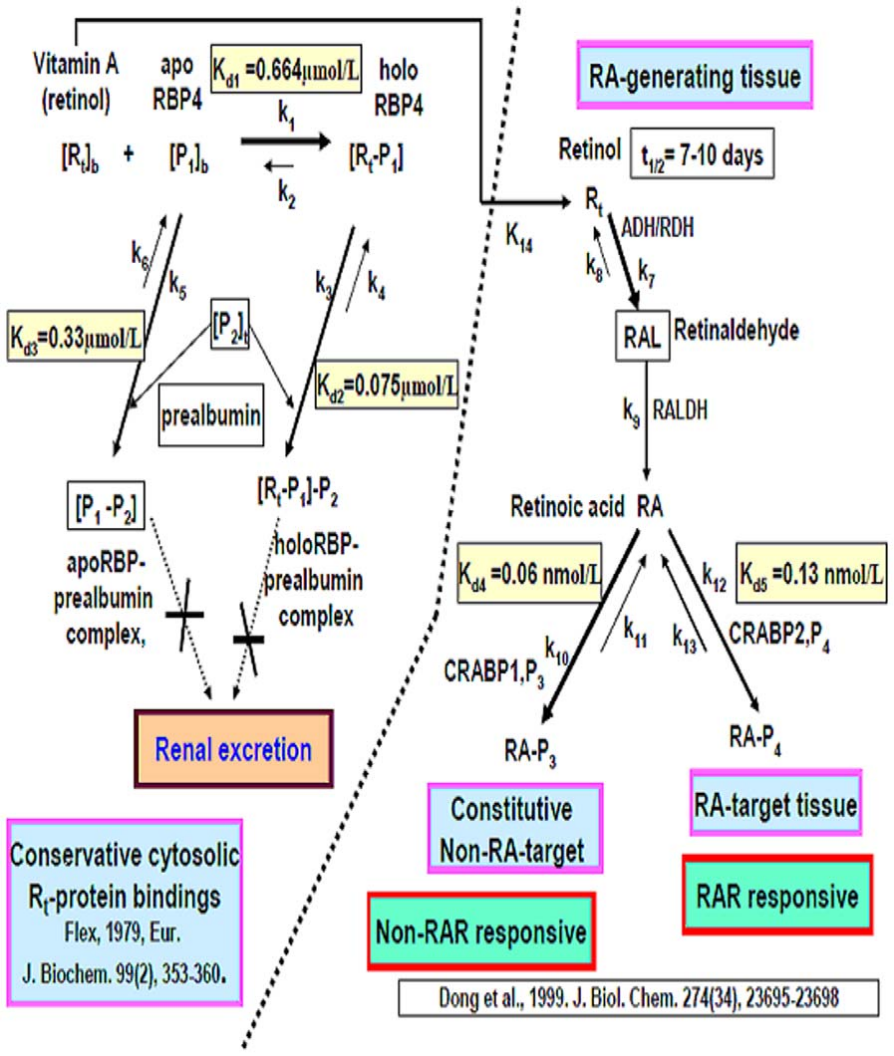
The Retinol-Retinoic Acid Homeostatic System.

RBP4 is assumed to be one of the factors controlling the local concentration of retinol [36]. In order to interpret how kinetic homeostatic control of retinol occurs, Fig. 4 shows the complex relationships between vitamin A, RBP4 and prealbumin in the cytosol and how this affects the generation of retinoic acid (RA) in RA generating tissues, and the binding of RA to retinoic acid receptor (RAR) in the RA target tissues (Fig. 4). RA biosynthesis in vertebrates occurs in two consecutive steps, these are the oxidation of retinol to retinal (retinaldehyde), which is followed by the oxidation of retinaldehyde to RA (Fig. 4). Enzymes of the MDR (medium-chain dehydrogenase/reductase; ADH alcohol dehydrogenase), SDR (shortchain dehydrogenase/reductase) and AKR (aldo-keto reductase) superfamilies have been reported to catalyze the conversion of retinol to retinaldehyde [37]. A model involving various RARs and tissue specific Hox gene expression has been proposed to explain the HDAC related effects during embryo development [14].

The estimated half life and the metabolic time of vitamin A (retinol) in the rat liver is 7 to 10 days [38]. In blood, RBP4 (P_1_) carries retinol (vitamin A, R_t_) in an equimolar ratio as [R_t_−P_1_] [39], [40] (Eq. 1) (K_1_ in Fig. 4). The complex further bind with prealbumin (transthyretin, TTR) (P_2_) to form the holoRBP prealbumin complex [R_t_−P_1_]−P_2_ (Eq. 2) (K_3_ in Fig. 4), and the major part of circulating RBP4 is found in the form of a complex with P_2_, namely the apoRBP-prealbumin complex [P_1_−P_2_] (Eq. 3) (K_5_ in Fig. 4) [39]. This prevents the loss of R_t_ and RBP through renal glomerular filtration. In reality only a very small amount of free RBP4 (P_1_) can be found in serum (the reverse reaction K_2_ of Eq. 1) [40] (Fig. 4).
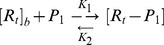
(1)

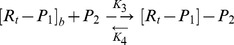
(2)

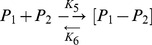
(3)

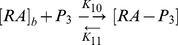
(4)

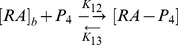
(5)


### The reversibility of retinol to retinaldehyde reaction is almost negligible

Although retinal can be reversibly reduced to produce retinol or irreversibly oxidized to produce RA [41], the reversible reaction always is almost negligible [42] (K8 in Fig. 4). Retinaldehyde reduction utilizes either NADH or NADPH as a cofactor, with NADH being the slightly more effective of the two [42].

### Retinoic acid conjugates with constitutive CRABP1 in non-RA target tissues, or alternatively with CRABP2 to form a RAR-responsive complex

Cellular RA binding protein 1 (CRABP1, P_3_) binds to RA to form a constitutive non-RA targeting complex [RA-P_3_] (Eq. 4); when cellular RA is complexed with CRABP2, it forms a RAR responsive conjugate [RA-P_4_] [43] (Eq. 5) (Fig. 4).
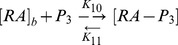
(4)

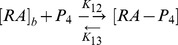
(5)


Dong et al. reported that the Kd values for the interaction of RA with the CRABPs to be 0.06 nmol/L and 0.13 nmol/L for CRABP-I (P_3_) and CRABP-II (P_4_), respectively [43], which suggests that most free RA comes from the dissociation of RA-P4 in RAR responsive tissue (Fig. 4). Other studies have indicated that CRABP is present in chick embryos at a constant level between stage 20 and stage 35 and the apparent K_d_ is 140–280 nmol/L [44].

### Downregulation of RBP4 tends to induce hypervitaminosis A

Table 4 lists the parameters used for the kinetic analysis. From Eq. 2 we have
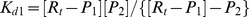
(6)


**Table 4:** Kinetic parameters used for calculating the effect of the downregulation of RBP4.

Parameter	Dimension/unit	Localization	Reference
Retinol R_t_, MW	286 kDa	Liver cytoplasm	
[R_t_]	1.73±0.34 µmol/L; 343.1±12.74 µg/L ( = 1.20 µmol/L)	Human cytoplasm; human serum	Flex et al., 1979; Raza et al., 2009.
RBP 4 (P_1_), MW	21000 kDa	Liver cytoplasm	Kanai et al., 1968.
[P_1_]	3.23±0.62 µmol/L	Human cytoplasm	Flex et al., 1979.
Prealbumin (P_2_), MW	55000 kDa (transthyretin, TTR)	Plasma	
[P_2_]	4.91±1.27 µmol/L	Human cytoplasm	Flex et al., 1979.
[R_t_−P_1_]−P_2_, MW	55286 kDa	Cytoplsam	This paper
K_d1_	0.664 µmol/L; 0.20 µmol/L	Cytoplasma; recombinant protein	This paper; Wysocka-Kapcinska et al., 2010
K_d2_	0.075±0.015 µmol/L	Plasma	Flex et al., 1979.
K_d3_	0.33±0.11 µmol/L; 0.4 µmol/L	Plasma; recombinant TTR	Flex et al., 1979; Berni et al., 1994.
K_d4_	0.06 µmol/L		Dong et al., 1999.
K_d5_	0.13 µmol/L		Dong et al., 1999.

By neglecting the free unbound retinol [40], we have [R_t_−P_1_]≈[R_t_] = 1.73 µmol/L, and [[R_t_−P_1_]−P_2_]≈[R_t_−P_1_] =  [1.73 µmol/L], and given K_d1_ = 0.075 µmol/L[39]. On substitution of these values into Eq. 6, this gives
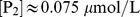



Thus the residual prealbumin P_2_ is [P_2_]_residual_ = [P_2_]_total_−[P_2_] = 4.91 µmol/L−0.075 µmol/L = 4.835 µmol/L, which is the free [P_2_] available for binding with P_1_. If [P_1_]_total_ = 3.23 µmol/L, then the maximum concentration of [P_1_−P_2_] attainable is only 3.23 µmol/L. The difference is the free P_2_, which is 4.835 µmol/L−3.23 µmol/L = 1.605 µmol/L. Given that K_d2_ = 0.33 µmol/L [39], then substitution of these values into Eq. 7, we gives
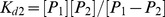
(7)

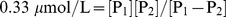


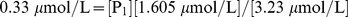



Thus the free [P_1_] that is not bound to or dissociated from [P_1_−P_2_] is 0.664 µmol/L.

Eq. 1 gives the dissociation equation:
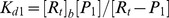
(8)


As given above: [R_t_−P_1_]≈1.73 µmol/L, [R_t_] = 1.73 µmol/L; and [P_1_] obtained from the above calculation, [P_1_] = 0.664 µmol/L:
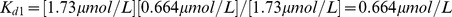
(9)


Wysocka-Kapcinska reported that apparent dissociation constant of retinol in relation to the recombinant protein is 2×10^−7^ M (i.e. 0.20 µmol/L), which is consistent with the published data for the native human protein [45].

The two independent and equivalent RBP binding sites on recombinant transthyretin (TTR) are characterized by a dissociation constant of about 0.400 µmol/L [46]. The values K_d1_ = 0.664 µmol/L obtained in this paper (Table 4) and equations 10 and 11 can then be used to calculate the free retinol levels [R_t_]_f_ based on Eq. 1 and Eq. 8. The results are listed in Table 5:
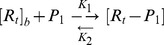
(10)

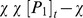


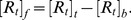
(11)


**Table 5:** The unbound cytoplasmic free retinol concentration is affected by differences in the level of RBP4.

%RBP4 level = %[P1]	Absolute cytoplasmic level (µmol/L)		
	Total RBP4, [P1]t	[Rt]b, calculated^a^	[Rt]f, calculated^a^
+50%	4.85	1.50	0.23
+40%	4.52	1.43	0.30
+30%	4.20	1.37	0.36
Normal level	3.23±0.62 (Flex, 1979)	1.17	0.56
−30%	2.26	0.95	0.78
−40%	1.94	0.85	0.88
−50%	1.62	0.75	0.98

a Calculated by Eq. 10. Kd1 = 0.664 µmol/L = [Rt]b[P1]/[Rt−P1] = [Rt]b[P1]/[P1]t−x].

Total retinol, [Rt]t = 1.73±0.34 µmol/L.

Based on the above, it is clear that any downregulation of RBP4 will tends to result in hypervitaminosis A. We have showed VPA causes downregulation of RBP4 by 32% (Table 2), which should induce a 39.3% increase in hypervitaminosis A (Table 5).

### Hypervitaminosis A tends to disrupt normal gene expression profile

It is likely that this highly raised level of unbound free retinol might have two possible effects. The first is direct teratogenicity, while the other is indirect teratogenesis after transformation of the retinol to retinoic acid [47]. In the normal state, RBP4 does not manifest its toxic effect unless the binding capacity is exceeded [39].

Vitamin A (retinol, VA) has a key role in vertebrate morphogenesis and is involved in determining body patterning and growth via control of cell proliferation and a range of other differentiation processes [48]. Hypervitaminosis A is likely to ultimately cause specific gene expression disruption at critical development stages [48]. A recent report has indicated that a high dietary intake of retinol can lead to bone marrow hypoxia and diaphyseal endosteal mineralization in rats [49]. Several reports have documented cognitive and behavioral deficits in the offspring of animals exposed to maternal hypervitaminosis A [50] as well as the presence of congenital anomalies in rats [51]. Conversely, upregulation of RBP4 may induce hypovitaminosis A. A deficiency in endogenous vitamin A may lead to local CNS positional cell apoptosis and blocking of the secretion of the binding protein that should be transported to epidermal cells during embryonic development [52].

### Hypervitaminosis A may simultaneously induce hyperretinoic acidosis

Hypervitaminosis A would seem to favor retinol flux into RA generating tissue (reaction K_14_), accelerating the oxidation of retinol; this will lead to a high concentration of RA (reactions K_7_ and K_9_) (Fig. 4). The biosynthesis of all trans-RA from all trans-retinol is catalyzed by human p-450 cytochromes. The Km and Vmax values for the NADP-dependent reaction are 19.4 mM and 52 pmol/min/mg protein (r^2^ = 0.77), respectively and those for NAD-dependent reaction are 41.5 mM and 280 pmol/min/mg protein (r^2^ = 0.96), respectively [53]. At low retinol levels, the NADP-dependent reaction will dominate, while the NAD-dependent oxidative conversion from retinol to RA is favored at high retinol levels [42].

Hypervitaminosis A during early development disrupts normal gene expression of the genes involved in RA signaling, disrupts normal gene expression of several genes that encode ECM proteins linked to skeletogenesis, such as *bglap* and *mgp* and affects VA homeostasis (RBP) [48]. RA, an analogue of vitamin A, is known to be teratogenic in laboratory animals and in reality has been implicated in a limited number of clinical case reports, including elective abortions (95/154), spontaneous abortions (26/154), and 21 malformed infants (21/154) [47]. The distribution pattern of cellular RA binding protein (CRABP) transcript shows a good correlation with the known target tissues for excess retinoid-induced teratogenesis (migrating primary mesenchyme and neural crest cells of the preoptic hindbrain), which suggests that cells expressing CRABP are those that cannot tolerate high levels of RA during their normal development [54].

In essence, investigating the role of many teratogenic medicines remains challenging, particularly in the context of developing a preventive strategy. To our beliefs, we are the first who has showed the application of kinetic analysis to predict the outcome when RBP4 is to be severely downregulated by VPA. The result clearly predicts that significant downregulation of RBP4 can cause hypervitaminosis A and hyperretinoic acidosis, which probably may result in a certain degree of teratogenicity. Whether the downregulation of RBP4 is a direct or an indirect mechanism to induce valproic acid tetragenicity still remains unclear. Suggestively, VPA therapy should be at the same time prescribed on an appropriate amount of vitamin A antagonist.
